# Network analysis of postpartum depression symptoms and perinatal risk factors in Chinese women: a prospective observational study

**DOI:** 10.3389/fpsyg.2025.1604013

**Published:** 2025-11-03

**Authors:** Xian Zhang, Jiasi Yao, Hongxiao He, Jiahe Li, Junying Li, Hong Lu, Xiaona Huang, Xiaobo Tian, Junxiao Liang, Luxia Gong, Ruyan Pang, Qiong Luo, Jinbing An, Xiu Zhu

**Affiliations:** ^1^School of Nursing and Health, Zhengzhou University, Zhengzhou, Henan, China; ^2^School of Nursing, Hebei Medical University, Shijiazhuang, Hebei, China; ^3^School of Nursing, Peking University, Beijing, China; ^4^Handan Vocational College of Science and Technology, Handan, Hebei, China; ^5^Child Health and Development Section, United Nations Children's Fund China Office, Beijing, China; ^6^Chinese Maternal and Child Health Association, Beijing, China; ^7^Department of Health Informatics and Management, School of Health Humanities, Peking University, Beijing, China

**Keywords:** postpartum depression, Edinburgh Postnatal Depression Scale, breastfeeding, network analysis, random forest, Bayesian network, perinatal risk factors

## Abstract

**Background:**

Postpartum depression (PPD) is a prevalent mental health issue with significant implications for maternal and infant wellbeing. Despite extensive research, the complex interplay of perinatal factors contributing to PPD remains inadequately characterized. This study utilizes a network analysis approach to identify central depressive symptoms, critical perinatal risk factors, and pathways contributing to PPD among Chinese postpartum women.

**Methods:**

A prospective observational study enrolled 377 women from 10 tertiary hospitals in China. Sociodemographic and perinatal factors were assessed shortly after childbirth. At 4–6 weeks postpartum, PPD symptoms were evaluated using the Edinburgh Postnatal Depression Scale (EPDS). Maternal and infant health outcomes, breastfeeding experiences, and family support were captured through self-administered questionnaires. Random forest and Bayesian network analyses were employed to identify influential factors and their interrelationships.

**Results:**

Among the participants, 98 women (26.0, 95% CI: 21.6–30.7%) screened positive for PPD (EPDS score ≥10). *Anxiety/Worry* emerged as the most central symptom in the network, with the highest strength and centrality (Degree = 0.893, Betweenness = 0.888). Key risk factors included breastfeeding challenges (RF = 0.752), maternal physical complaints (RF = 0.431), and adverse infant health conditions (RF = 0.350). Protective effects were observed for prolonged skin-to-skin contact, family support for breastfeeding, and positive childbirth self-perception. Hospital type served as a central bridging node within the network, strongly connected with factors related to peripartum care services, childbirth experience and PPD.

**Conclusion:**

This study reveals a significant prevalence of PPD among mothers in China. It underscores the positive impact of skin-to-skin contact, continuous postpartum care and tailored breastfeeding support in addressing PPD. These findings will advance PPD prevention strategies and inform evidence-based clinical practice in postpartum care.

## 1 Introduction

Postpartum depression (PPD), the most common childbirth complication, has received increasing clinical, public and scientific attention. Characterized by persistent sadness, anxiety, insomnia, disorganized behavior and irritability, PPD typically manifests within 4–6 weeks postpartum but can endure for months or years (American Psychiatric Association (APA), 2013). Globally, PPD affects approximately 17.2% (95% CI: 16.0–18.5%) of postpartum women (Wang et al., 2021) and 23.1% (95% CI: 21.8–24.5%) in low- and middle-income countries (LMICs) (Roddy et al., 2023). Various studies demonstrate that maternal PPD is a significant issue that can steal motherhood, and has a range of effects on the health of the entire family (Ahmadinezhad et al., 2024; Dagher et al., 2020). In fact, PPD can negatively affect the couple’s and parent–child relationship, and increase the risk of adverse infant health outcomes (Dadi et al., 2020) as well as offspring’s physical, behavioral and cognitive developmental outcomes (Gelaye et al., 2016; Sridhar et al., 2025), resulting in substantial humanistic and economic burden affecting the whole society (Margiotta et al., 2022; Moore Simas et al., 2019).

The pathophysiology and etiologies of PPD are complex, multifactorial, and, in most cases, incompletely elucidated (American College of Obstetricians and Gynecologists (ACOG), 2023). Alongside socio-demographic and biological factors, recently many evidences focused on the birth-related contextual variables that constitute significant risk factors for PPD development (Coo et al., 2023; Rados et al., 2022). Results from Grekin et al. (2021) indicated both subjective experiences of childbirth (including perception of support from professionals or others, feelings of control and security) and objective birth characteristics (like childbirth interventions and infant outcomes) were robustly associated with symptoms of posttraumatic stress and depression, supported by relevant evidence that a lower level of satisfaction with childbirth was a significant predictor of a higher risk of PPD (Coo et al., 2023). All these findings strongly suggested that high-quality peripartum care and a positive birth experience can make the psychological adaptation in the early postpartum period easier and have long-lasting effects on maternal wellbeing.

Common health problems or negative experience in the early postpartum period were significant risk factors of PPD. Specifically, the quality of maternal care and support following childbirth plays a crucial role, as it is essential in helping women transition into motherhood and cope with its associated challenges (Silva-Fernandez et al., 2023). Furthermore, a systematic review of infant health outcomes in LMICs suggested adverse infant health outcomes were associated with an increased risk of PPD (Dadi et al., 2020). Negative breastfeeding experiences, e.g., breastfeeding challenges, discomforts, and lack of breastfeeding supports were also reported to precede the onset of depressive symptomatology (Tanganhito et al., 2020; Yuen et al., 2022). Conversely, exclusive breastfeeding may exert a protective effect against PPD (Lubis et al., 2024; Shimao et al., 2021). While previous studies have provided valuable insights into the risk and protective factors for PPD development and persistence, these correlates are often examined in isolation. However, pregnancy, childbirth, and the postpartum period represent interconnected experiences that likely interact to shape maternal emotional and biological vulnerabilities, culminating in conditions such as PPD.

Due to the complex interaction among related variables, standard regression models or parametric statistical methods can hardly express such relationships (generally nonlinear) (Arora et al., 2019; Xu et al., 2018). In contrast, some non-parametric methods such as random forest or other machine learning approaches can be employed to evaluate the effects of risk factors on a given response variable and identify critical factors (Cellini et al., 2022; Zhao et al., 2024). Furthermore, Bayesian network can be used to explore direct and indirect effects among sets of factors, which will provide clues for some potential intervention strategies, such as certain pathways or action (Arora et al., 2019; Lazarov et al., 2020). On the other hand, based on the network structure, we can also identify local structures by community detection methods (Rajeh and Cherifi, 2022), which can help to explore candidate intervention strategies for specific targets and improve health outcomes. However, few studies focused on the system structure and correlates of PPD using network approaches. Therefore, to gain a more comprehensive understanding of the complex nature of how these perinatal and postpartum factors are interconnected, it might be more appropriate to take a network approach.

This prospective observational study examined the prevalence and network structure of postpartum depressive symptoms, and also applied random forest and Bayesian network methods to identify critical perinatal and postpartum factors and pathways influencing PPD among Chinese women. Our findings aim to inform targeted, system-level interventions to mitigate PPD risk and enhance maternal–infant health outcomes.

## 2 Materials and methods

### 2.1 Study setting and participants

This study was conducted in 10 tertiary hospitals (three general hospitals and seven maternal & child hospitals) in nine provinces spanning eastern, central, and western regions of mainland China. We selected these nine provinces to reflect diverse socioeconomic development levels. Participants were systematically recruited from Maternity Departments of the selected hospitals between September and October 2022. Eligible criteria included: (a) age ≥ 18 years; (b) vaginal delivery, (c) singleton, full-term birth (gestational age ≥ 37 weeks); (d) absence of serious pregnancy complications or infectious diseases. The exclusion criteria included: (a) neonatal admission to the Neonatal Intensive Care Unit (NICU) at birth; (b) women with a psychiatric disorder or intellectual disability impairing normal communication.

**Figure 1:**
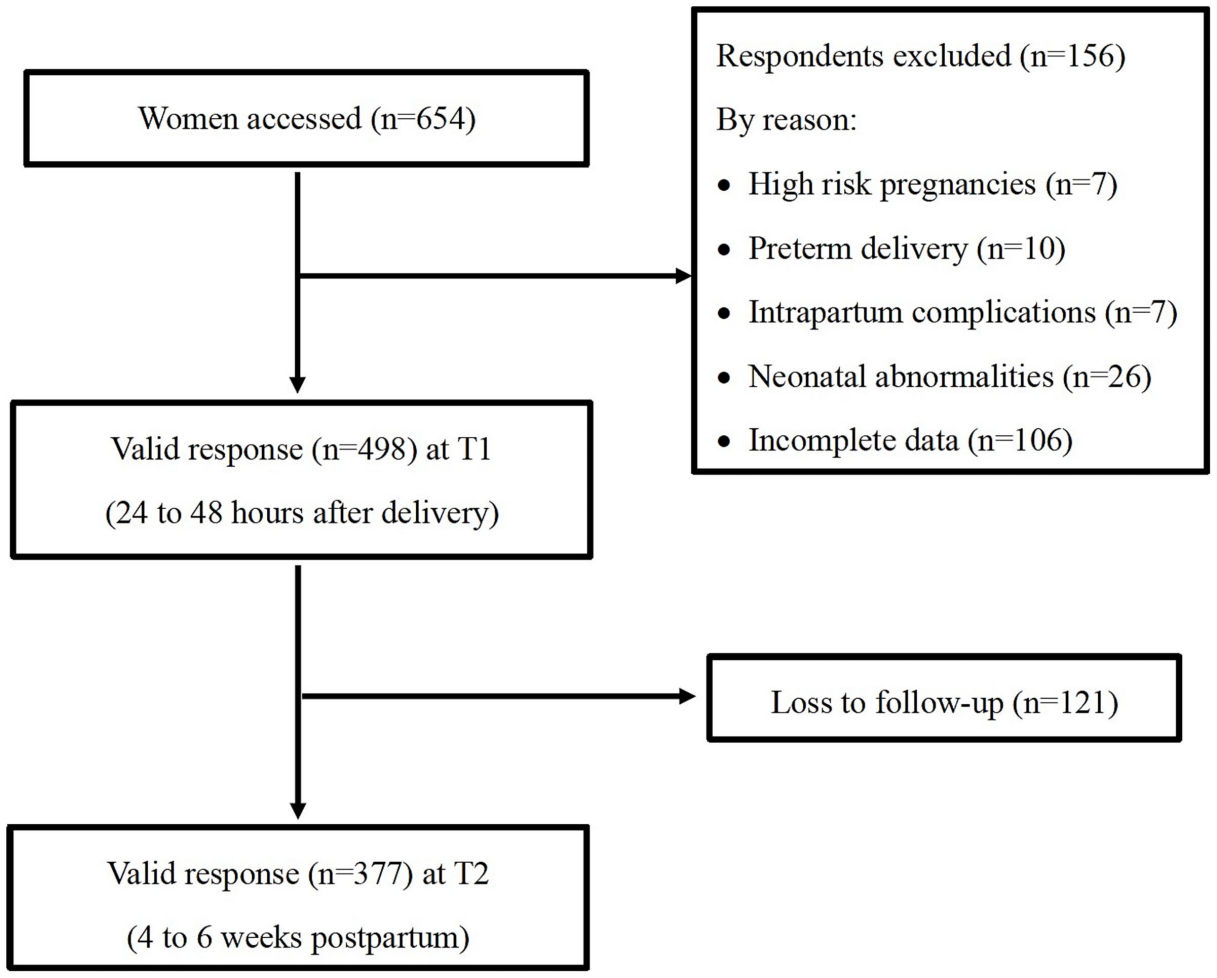
Flow chart of participants’ recruitment process.

The sample size was determined to estimate the prevalence of PPD with ±4.0% precision at a 95% confidence level (two-sided *α* = 0.05). The expected prevalence was derived from a systematic review by wang et al. (2021), which gave an overall PPD prevalence of 18.0% among Chinese women. This yielded a minimum sample size of 354 participants. Accounting for an anticipated 25% attrition rate, as often seen with longitudinal online surveys, the final sample size was adjusted to 472 participants. Using a systematic sampling method, and, guided by the large-sample theory (Lehmann, 1999), a minimum of 30 participants in each hospital were enrolled. Of 654 women initially accessed, 156 were excluded due to ineligibility or incomplete baseline data. This yielded 498 participants at baseline (24–48 h after delivery), with 377 completing follow-up assessments at 4 to 6 weeks postpartum (see Supplementary Table 1 for the number of participants in each hospital), reaching a follow-up rate of 75.7% (Figure 1). Comparative analysis revealed no significant differences in demographic or obstetric characteristics (except mode of delivery) between retained participants in this study and those lost to follow-up (*n* = 121). This study was approved by the Peking University Institutional Review Board (Approval No. IRB00001052-22047).

### 2.2 Measures

Data collection tools were adapted from the WHO’s Record Form for Censorship (World Health Organization, 2016) and tailored to China’s midwifery context (Yan et al., 2021). Baseline data (T1: 24–48 h postpartum) included sociodemographic characteristics, and perinatal factors (pregnancy, birth and early newborn care practices) via self-administered questionnaires and birth records reviewed by nurse-midwives. Follow up data (T2: 4–6 weeks postpartum) assessed maternal and infant health, breastfeeding experiences, family support, and postpartum depression via self-administered questionnaires.

#### 2.2.1 Sociodemographic information

The sociodemographic information included participants’ age, parity, education, employment status, annual household income, baby’s gender, maternity leave duration, and primary postpartum caregiver.

#### 2.2.2 Perinatal measures

Pregnancy-related factors, including prenatal education attendance, regularity of prenatal checkups (self-reported) and pregnancy complications (extracted from medical records).

Birth-related factors, including mode of delivery (spontaneous vaginal or instrumental), pain-relief methods, labor companionship (except medical staff), labor augmentation, induced labor, episiotomy, and blood loss (from medical records).

Childbirth experience was measured by the self-report Chinese version of the Childbirth Experience Questionnaire (CEQ-C) (Zhu et al., 2019). It is a comprehensive tool first developed in Sweden in 2010 (Dencker et al., 2010) and has been confirmed to be valid and reliable by various studies (Nilver et al., 2017; Soriano-Vidal et al., 2016). The Chinese version consists of 19 items covering four domains: professional support, own capacity, self-perception, and participation. Each item was scored on a 4-point Likert scale (1 = totally disagree, 4 = totally agree), with higher scores indicating more positive childbirth experiences. The CEQ-C has good validity and reliability, with an internal consistency of 0.88 and a good model fit (Zhu et al., 2019).

Early newborn care practices, including duration of skin-to-skin contact (SSC: 0, <30, 30–59, 60–89, ≥ 90 min), early breastfeeding initiation (within 1 h postpartum) and rooming-in, were collected through maternal self-report.

#### 2.2.3 Postpartum measures

Maternal health outcomes: Self-reported health-seeking behaviors (seeking help from medical professionals due to maternal health concerns) during the 4–6 weeks postpartum period and the occurrence of common maternal health problems, including prolonged lochia, abnormal perineum incisions and lactational mastitis.

Infant health outcomes: Self-reported occurrence of common newborn or infant infection symptoms, including cord stump infection symptoms (red and swollen umbilical region, blood oozing and fluid oozing), eye infection symptoms (redness around newborn’s eyes and eye discharge), respiratory system infection symptoms (symptoms like coughing and runny nose, or diagnosed tracheitis, bronchitis or pneumonia), diarrhea and other common infection symptoms. Infants with any of these symptoms were categorized as having common infant health problems.

Breastfeeding experience: Breastfeeding pattern was assessed by “In the past 24 h, have you added any other foods (such as water or solid food) to your child in addition to breast milk?” Exclusive breastfeeding meant that infants were fed only with breast milk without any liquids or solid food, except for prescribed medicines, oral rehydration solution, vitamins and minerals (WHO and UNICEF, 2021). We also collected information about whether they had breastfeeding challenges or not, including self-perceived insufficient milk, not knowing when to breastfeed, sore nipples when breastfeeding, and distending pain in the breast.

Family support was assessed via the Breastfeeding Family Support Questionnaire. It is a 9-item scale developed by Zhu et al. (2013) and has two dimensions: ‘behavior support’ and ‘psychological support’. This scale is scored on a 4-point Likert scale (1 = completely disagree, 4 = completely agree), and higher scores reflect stronger breastfeeding family support. It has also demonstrated good psychometric properties. The reliability of the questionnaire was high as its Cronbach’s alpha was 0.886.

Postpartum depression was assessed by the Chinese version of the Edinburgh Postnatal Depression Scale (EPDS) (Lee et al., 1998). Responses are scored along a 4-point scale from 0 to 3 with a minimum total score of 0 and a maximum total score of 30. Higher scores indicate more depressive symptoms. The prevalence of PPD was identified by using the recommended cut-off point of 10 or above (Liang et al., 2020).

### 2.3 Data collection procedure

Eligible participants were enrolled by trained nurse-midwives. After obtaining informed consent, detailed instructions were provided to participants. At T1 (24–48 h postpartum), participants scanned a QR Code via WeChat to access an electronic questionnaire capturing participant’s demographic data, early newborn care practices, and childbirth experience. Nurse-midwives also reviewed medical records to extract pregnancy complications and birth-related clinical data. At T2 (24–48 h postpartum), a follow-up questionnaire was distributed through WeChat to collect data related to maternal and infant health information, breastfeeding experiences, family support and PPD symptoms.

### 2.4 Data analysis

Data were analyzed using SPSS 20.0 and R software 4.4.1. Means and standard deviations (SD) were used to describe continuous variables, while Mann–Whitney U test and Kruskal-Walls H test were used to identify the differences. The number (*n*) and percentage (%) were used to describe categorical data, and the chi-square test and Fisher’s exact test were used to identify differences. Moreover, Spearman correlations examined the relationships between the EPDS score and other continuous variables (childbirth experience and breastfeeding family support). Finally, Random Forest algorithm was used to obtain the critical risk factors on PPD based on R package of “random Forest,” and Bayesian network was performed to explore the relationship among related factors based on R package of “bnlearn.” Furthermore, to obtain a stable Bayesian network, a bootstrap method was employed with 1,000 iterations, and the edges appeared in at least 50% of the bootstrap iterations were included in the final averaged Bayesian network (Lazarov et al., 2020). By utilizing the network structure along with important nodes and edges, we attempted to explore the potential intervention targets.

## 3 Results

### 3.1 Participant demographic and perinatal characteristics

Table 1 summarizes the demographic and perinatal characteristics of the 377 women who completed the follow up survey at 4–6 weeks postpartum. Among them, 118 (31.3%) participants were recruited from general hospitals and 259 (68.7%) were from maternal & child health (MCH) hospitals. The participants were 21 to 41 years old (mean = 30.59, SD = 4.02), with 63.9% holding a bachelor’s degree or above.

**Table 1:** Participant characteristics (*n* = 377).

Variable	*n* (%)/Mean (SD)
Demographics	
Age group	
<35	311 (82.5)
≥35	66 (17.5)
Education level	
Junior college and below	136 (36.1)
Bachelor or above	241 (63.9)
Employment status	
Paid work	232 (61.5)
Unemployed	145 (38.5)
Annual household income	
<120,000 yuan	128 (34.0)
120,000–300,000 yuan	155 (41.1)
>300,000 yuan	94 (24.9)
Parity	
Primipara	239 (63.4)
Multipara	138 (36.6)
Pregnancy-related	
Pregnancy complication	
Yes	195 (51.7)
No	182 (48.3)
Prenatal education	
Yes	134 (35.5)
No	243 (64.5)
Prenatal checkups	
Regular	342 (90.7)
Irregular	35 (9.3)
Birth-related	
Mode of delivery	
Spontaneous vaginal	372 (98.7)
Instrumental	5 (1.3)
Pain management	
Pharmacological pain relief	257 (68.2)
Non-pharmacological pain relief	245 (65.0)
No pain relief method	53 (14.1)
Mean CEQ-C score	3.24 ± 0.48
Professional support	3.64 ± 0.49
Own capacity	3.25 ± 0.64
Self-perception	2.74 ± 0.68
Participation	3.08 ± 0.78
Early newborn care practice	
Duration of SSC (min)	
No SSC	63 (16.7)
<30 min	154 (40.9)
30–59 min	65 (17.2)
60–89 min	21 (5.6)
≥90 min	74 (19.6)
Initiation of the first breastfeeding	
Within 1 h after birth	298 (79.0)
After 1 h	79 (21.0)
Rooming in	
Yes	364 (96.6)
No	13 (3.4)

CEQ-C, Chinese version of the Childbirth Experience Questionnaire; SSC, Skin-to-Skin Contact.

Most participants (63.4%) were primiparous. Nearly all (98.7%) underwent spontaneous vaginal delivery, with 24.4% receiving labor induction, 34.7% receiving labor augmentation, and 23.3% having episiotomy. About two thirds (68.2%) of the participants were administered pain relief medication, whereas 65.0% had received non-pharmacological pain relief. Post-delivery, 16.7% (*n* = 63) of mother-newborn pairs had no SSC, while only 19.6% (*n* = 74) women reported SSC duration exceeding 90 min. Early breastfeeding initiation was reported by 79% (*n* = 298). Childbirth experiences, assessed via CEQ-C, yielded a mean score of 3.24 (SD = 0.48; range 1.42–4.00). Detailed demographic and perinatal data are provided in Supplementary Table 2.

### 3.2 Maternal and infant health outcomes, breastfeeding experience and family support

During the 4–6 weeks postpartum period, 22.8% of participants (*n* = 86) reported engaging in health-seeking behaviors for maternal health concerns, while 43.2% (*n* = 163) experienced breastfeeding challenges. Among infants, 26.3% (*n* = 99) exhibited common health issues, with eye infection symptoms (11.7%) and diarrhea symptoms (11.1%) being the most prevalent. At 4–6 weeks postpartum, the exclusive breastfeeding rate was 54.4%. The breastfeeding family support was at a moderate to high level with a mean score of 28.81 (SD = 4.01; range 18–36).

### 3.3 Prevalence and network structure of postpartum depressive symptoms

**Figure 2:**
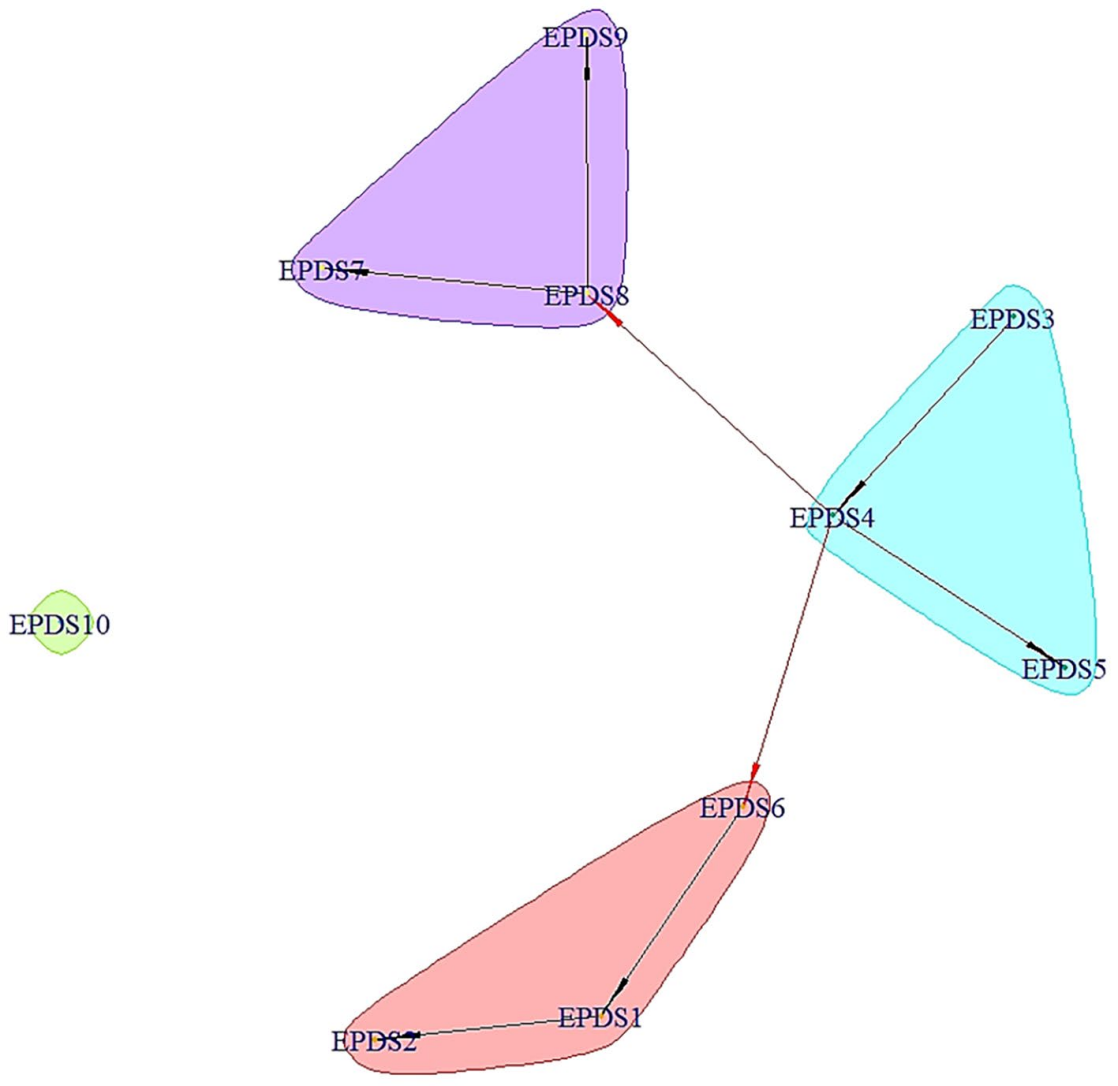
The network structure of postpartum depressive symptoms among Chinese mothers at 4–6 weeks postpartum. The red arrows represent important edges with strength above 0.7, while the black ones represent edges with strength of 0.7 or less. Abbreviations: EPDS 1, pleasure loss; EPDS 2, pessimism; EPDS 3, self-blame; EPDS 4, anxiety/worry; EPDS 5, panic; EPDS 6, overwhelmed; EPDS 7, insomnia; EPDS 8, sad mood; EPDS 9, crying; EPDS 10, self-harm ideation.

The mean EPDS score was 6.12 (SD = 5.50, range: 0–25). A total of 26.0% (*n* = 98, 95% CI: 21.6–30.7%) screened positive for PPD (EPDS ≥10). Figure 2 illustrates the network structure of depressive symptoms. *Anxiety/worry* (EPDS item 4) emerged as the most central symptom, exhibiting the highest strength (Degree = 0.893, Betweenness = 0.888), which connected to 40% of the symptoms, including *Sad Mood*, *Overwhelmed*, *Self-blame*, and *Panic*. *Sad mood* (EPDS 8) and *Overwhelmed* (EPDS 6) also occupied central positions within the network. In contrast, *Self-harm ideation* (EPDS 10) was relatively isolated in the network, showing no significant connections with other symptoms. See Supplementary Table 3 for complete centrality indices.

### 3.4 Complex network analysis of the relationships between PPD and related variables

#### 3.4.1 Influential factors of PPD ranked by random forest method

Using random forest modeling, we calculated the transformed random forest values (RF) of influential factors of maternal PPD. The top 10 influential factors were summarized in Table 2. Overall, breastfeeding challenges ranked the highest (RF = 0.752), suggesting its significant impact on PPD. Notably, three out of the top 10 factors were about maternal and infant physical health, including maternal physical complaints at early postpartum, common infant health problems and pregnancy complications. Meanwhile, SSC duration (RF = 0.650), family support for breastfeeding (psychological support: RF = 0.566; behavioral support: RF = 0.387), as well as childbirth self-perception (CEQ domain 3: RF = 0.300) also had strong connections with PPD.

**Table 2:** Importance ranking of influential factors for postpartum depression.

Ranking	Variables	Random forest (RF)
1	Breastfeeding challenges	0.752
2	Duration of skin-to-skin contact (SSC)	0.650
3	Psychological support for breastfeeding	0.566
4	Maternal physical complaints	0.431
5	Household registration	0.429
6	Behavioral support for breastfeeding	0.387
7	Infant health problems	0.350
8	Living place	0.340
9	Pregnancy complications	0.302
10	Self-perception (CEQ domain 3)	0.300

#### 3.4.2 Main pathways and communities of PPD and related factors in Bayesian network

**Figure 3:**
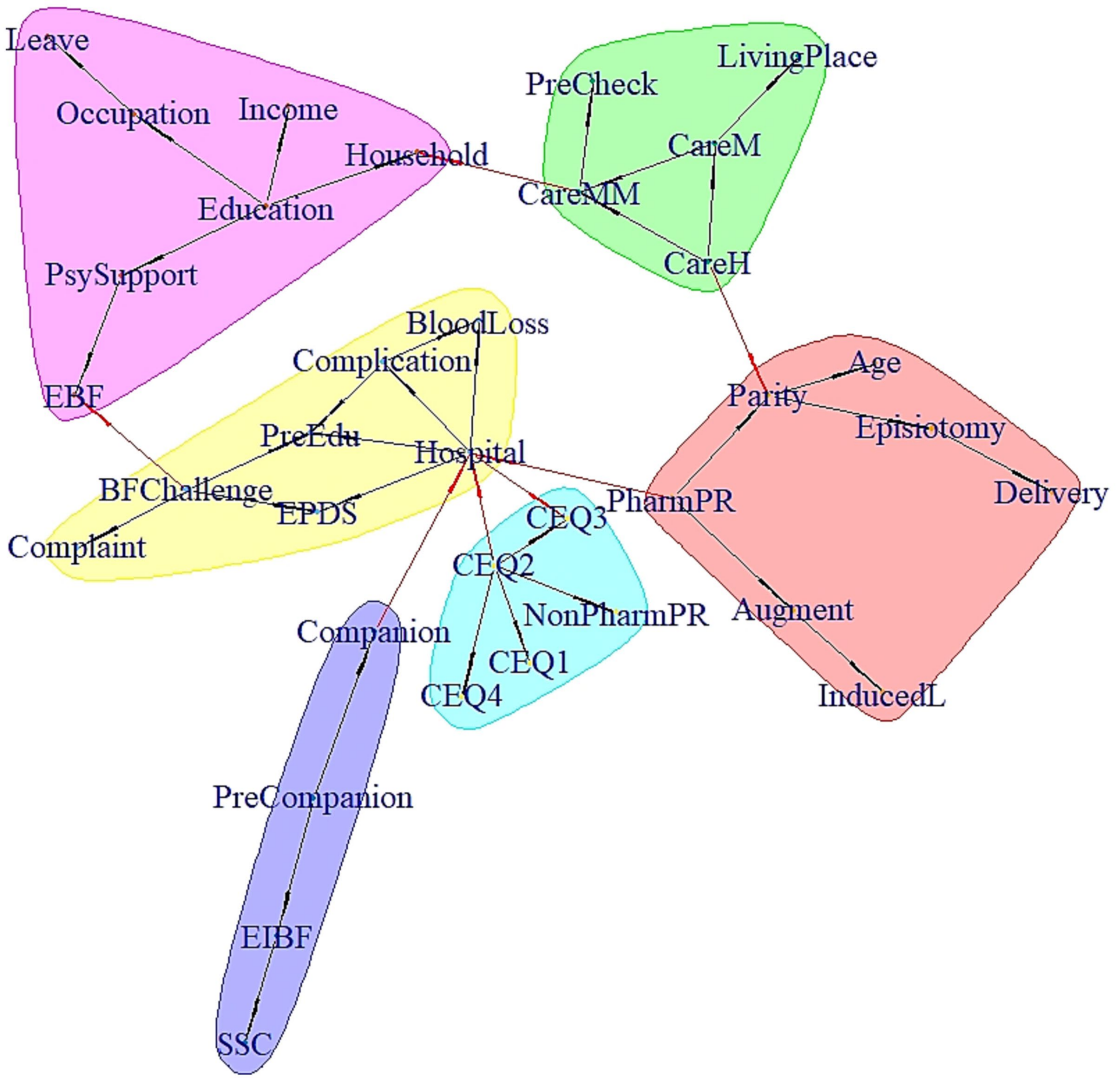
Pathways and communities of PPD and related factors. The red arrows represent important edges with strength above 0.7, while the black ones represent edges with strength of 0.7 or less. The full names of the abbreviations are as follows: Augment, Labor augmentation; BFchallenge, Breastfeeding challenges; CareH, Care by husband; CareM, Care by mother; CareMM, Care by maternity matron; CEQ, Childbirth Experience Questionnaire domain; Companion, Companionship during labor; Complaint, Maternal physical complaints; Complication, Pregnancy complications; Delivery, Mode of delivery; EBF, Exclusive breastfeeding; EIBF, Early initiation of breastfeeding; EPDS, Edinburgh Postnatal Depression Scale score≥10; Hospital, Type of hospital; Household, Household registration; Income, Family income; InducedL, Induced labor; Leave, Maternity leave; NonPharmPR, non-pharmacological pain relief; PharmPR, Pharmacological pain relief; PreCheck, Prenatal checkup; PreEdu, Prenatal education; PreCompanion, Prenatal companionship; PsySupport, Psychological support; SSC, Skin-to-skin contact duration.

Figure 3 illustrates the Bayesian network structure with sociodemographic characteristics, pregnancy- and birth-related factors, early newborn care practices, and postpartum factors associated with PPD. Overall, the relationships of breastfeeding challenges, maternal physical complaints, childbirth experience (CEQ domains), type of hospital, and PPD were among the strongest connections in the network. There were also strong links between psychological support from family, exclusive breastfeeding, prenatal education and breastfeeding challenges. Hospital type was centrally embedded in the network, and was strongly connected with CEQ domains, factors related to perinatal care services (prenatal education, pain relief methods, companionship during labor, SSC duration, etc.) and perinatal health conditions (pregnancy complication and postpartum blood loss). Among the four aspects of childbirth experience, self-perception (CEQ domain 3) and own capacity (CEQ domain 2) were key aspects that indirectly connected with PPD through the bridge of facility type. Most sociodemographic variable nodes (like age, parity, living place and maternity leave) were marginal, and indirectly or weakly linked to the central nodes.

The Bayesian network also identified some interesting communities among the variable nodes. Most notably, there was a birth experience-related community including childbirth experience (with Own Capacity centrally embedded in the four CEQ domains), and non-pharmacological pain relief methods. Another community was about factors related to early breastfeeding, including labor companionship, SSC duration, and early initiation of breastfeeding.

#### 3.4.3 Correlates of postpartum depression by univariate analyses

Based on the results of random forest methods, we further analyzed the relationships between the top 10 influential factors and the EPDS scores. As shown in Table 3, pregnancy complications, breastfeeding challenges, health seeking behaviors after discharge and baby with common infant health problems were all significantly correlated to higher EPDS scores (*p* < 0.05). However, no significant differences were found between women with different household registrations, living places at early postpartum and SSC durations (*p* > 0.05).

**Table 3:** Differences in EPDS scores among women with different characteristics.

Influential factors	*n* (%)	Total EPDS score M (SD)	*Z*/*H*	*p* value
Pregnancy complications			−2.155	0.031
Yes	195 (51.7)	6.82 (5.93)		
No	182 (48.3)	5.38 (4.92)		
Duration of SSC (min)			7.166	0.127
No SSC	63 (16.7)	7.02 (6.23)		
<30 min	154 (40.9)	6.12 (5.28)		
30–59 min	65 (17.2)	4.97 (5.01)		
60–89 min	21 (5.6)	4.05 (2.80)		
≥90 min	74 (19.6)	6.96 (6.06)		
Breastfeeding challenges			−6.048	<0.001
Yes	163 (43.2)	8.04 (5.76)		
No	214 (56.8)	4.66 (4.83)		
Health seeking behavior after discharge			−4.255	<0.001
Yes	86 (22.8)	8.51 (6.19)		
No	291 (77.2)	5.42 (5.08)		
Common infant health problems			−4.195	<0.001
Yes	99 (26.3)	8.15 (5.93)		
No	278 (73.7)	5.40 (5.16)		

The Mann–Whitney U test and Kruskal-Walls H test were performed, where the positive or negative value of *Z*/*H* indicates the relative size relationship between the medians of the sample groups.

The result of Spearman correlation analysis indicated breastfeeding family support was negatively related to levels of depressive symptoms (*r* = −0.272, *p* < 0.001). Interestingly, there was no statistically significant relationship between childbirth experience and EPDS scores (*p* > 0.05). However, when we split the data into two sets by hospital type (general/MCH hospitals), a negative relationship was found between childbirth experience and EPDS scores (*r* = −0.200, *p* = 0.001) among women in MCH hospitals.

Since our network analysis indicated hospital type was related to PPD, we further explored whether perinatal care practices, childbirth experiences, adverse health outcomes and EPDS scores differed based on hospital type. The results showed that women in MCH hospitals reported more depressive symptoms than those in general hospitals (mean EPDS: 6.90 vs. 4.42; *Z* = −4.023, *p* < 0.001). This difference may be driven by their elevated rates of pregnancy complications and adverse maternal/newborn outcomes (*p* < 0.05). As key referral centers in China, MCH hospitals are therefore more likely to serve women with complex or high-risk pregnancies, which may contribute to the observed differences in health outcomes and mental health status. Notably, the superior perinatal care in MCH hospitals—including better labor pain relief coverage, longer SSC durations, and higher level of childbirth experiences (*p* < 0.001)—may have attenuated depression levels that could otherwise have been even higher given their poorer health outcomes. Refer to Supplementary Table 4 for details.

## 4 Discussion

This prospective observational study applied random forest and Bayesian network to identify central depressive symptoms, critical risk factors, and the complex interplay of perinatal and postpartum factors associated with PPD among Chinese women following vaginal deliveries. By mapping symptom networks and variable interactions, our findings provide actionable insights for targeted interventions to mitigate PPD risk and optimize maternal mental health outcomes.

PPD prevalence was substantial in our cohort, with 26.0% of participants screening positive at 4–6 weeks postpartum. Network analysis identified *Anxiety/Worry* (EPDS 4) as the most central symptom, demonstrating the highest node strength and robust connections to *Sad Mood*, *Overwhelmed*, *Self-blame*, and *Panic*. This centrality underscores its pivotal role in the PPD symptom network and aligns with evidence suggesting anxiety symptoms may precede comorbid depressive conditions (Falah-Hassani et al., 2017; Phua et al., 2020). *Sad Mood* (EPDS 8) and *Overwhelmed* (EPDS 6) emerged as secondary central nodes, potentially reflecting distress driven by perceived challenges in meeting maternal demands during this critical adjustment period (Gao et al., 2010; Kristensen et al., 2018). Critically, targeting these central symptoms in clinical practice—through enhanced professional and family support to alleviate parenting stress and improve emotional regulation—may disrupt symptom deepening and mitigate PPD severity.

Breastfeeding challenges emerged as the predominant risk factor for PPD in our study, demonstrating the highest RF among all predictors. This finding aligns with systematic reviews linking breastfeeding challenges to adverse mental health outcomes (Yuen et al., 2022). Notably, 43.2% of women reported breastfeeding challenges, underscoring its clinical significance during the early postpartum period. Conversely, family support for breastfeeding (both psychological and behavioral support) constituted a key protective factor, a finding that underscores the fundamental role the family network plays in shaping mother’s breastfeeding self-efficacy and related outcomes (García-Fernández et al., 2023; Hu et al., 2025). This is also consistent with findings from a prospective cohort study in Canada (Chaput et al., 2016) that women’s perception of breastfeeding support significantly modified the primary association between breastfeeding difficulties and PPD. As highlighted in the Lancet Breastfeeding Series (Perez-Escamilla et al., 2023), negative breastfeeding experiences are primary reasons for prematurely stopping breastfeeding. Therefore, it is recommended to integrate family-centered education into prenatal and postnatal care, with clearly defined evidence-based support strategies to optimize breastfeeding experiences and reduce the risk of PPD.

Physical health conditions of both the mother and baby also play a key role in the occurrence of PPD. Women with physical problems and adverse infant outcomes are vulnerable to psychological disorders (Capik and Durmaz, 2018; Dadi et al., 2020), as the discomforts and increased stress due to poor health conditions can lead certain common symptoms (like fatigue and worry) into a network of depressive symptoms (Baez et al., 2021). Therefore, postpartum visits, remote counseling and ongoing postpartum care should be advocated to timely identify mother and baby’s health issues and provide services and support corresponding to each woman’s individual needs.

Regarding childbirth-related factors, SSC duration and childbirth experiences also stood out and shared indirect connections with PPD. While univariate analysis showed no significant EPDS score differences across SSC durations, random forest modeling identified SSC duration as a clinically relevant PPD predictor, a novel finding highlighting machine learning’s utility in detecting complex relationships obscured by traditional methods (Cellini et al., 2022). This aligns with WHO recommendations for early essential newborn care (EENC), which emphasize immediate and sustained SSC (World Health Organization, 2022). Previous evidence also suggested close physical contact with infants may activate the maternal oxytocinergic system that modulate attachment bond formation (Markova and Siposova, 2019), promote parenting self-efficacy and alleviate stress and anxiety (Huang et al., 2022). However, there are still divergent recommendations for SSC duration, and the evidence on the optimal duration of SSC for maximum benefits remains uncertain (Li et al., 2024). Further studies are necessary to confirm the psychological benefits of SSC for women and to explore the dose–response relations.

Crucially, women’s self-perception and own capacity involved in the whole childbirth process emerged as key PPD determinants, consistent with the study by Rados et al. (2022) that women’s personal attributes, including feeling anxiety or being in control during childbirth, have prolonged effect on postpartum mental wellbeing. The importance of women’s involvement in their own maternity care is well-established, with shared decision-making and autonomy being recognized as critical elements that positively impact the birth experience and overall satisfaction (World Health Organization, 2018; Deherder et al., 2022). Our finding that non-pharmacological pain relief was strongly connected with key aspects of the childbirth experience reinforces this, highlighting the importance of incorporating humanistic practices that offer women more choices. Therefore, to improve maternal mental health, it is imperative that healthcare institutions not only promote women’s active participation but also systematically monitor their perceptions and experiences of maternity care to guide quality improvement.

Hospital type served as a central bridging node within the network, strongly connected with factors related to peripartum care services, childbirth experience and PPD. However, its centrality likely reflects not the intrinsic properties of institutional classification, but rather the aggregation of systemic disparities—including socioeconomic stratification (parity, education level, household registration, etc.), differential clinical practices (labor pain management, SSC), and diverging maternal health trajectories (pregnancy complications, breastfeeding challenges, etc.)—that become embedded within specific hospital types. This pattern suggests that hospital type operates as a conduit for pre-existing vulnerabilities and varied care ecosystems, collectively shaping psychological outcomes. As the focus of China’s maternal and child healthcare has shifted from survival toward thriving (Qiao et al., 2021), our results underscore that institutional labels alone cannot drive progress. Policymakers must transcend hospital-centric paradigms by standardizing evidence-based, humanized practices (e.g., nonpharmacy pain relief, companion support, prolonged SSC) across all facilities, while simultaneously addressing upstream determinants that funnel vulnerability into specific care pathways. Only through this integrated approach—simultaneously standardizing high-quality perinatal care and addressing systemic inequities—can we effectively mitigate PPD risk and improve maternal psychological wellbeing.

### 4.1 Strengths and limitations

The present study is a new attempt to provide a network perspective on how a set of pregnancy- and birth-related variables as well as postpartum factors interacted and influence PPD. Moreover, we used a prospective longitudinal design and collected data over multiple time points based on their importance in clinical practices of multiple hospitals. The addition of intrapartum and early newborn care practices, hospital type and childbirth experiences as important nodes into the influential factor networks could be insightful, especially in combination with longitudinal data. In addition, we prepared detailed information in the questionnaires for participants to comprehensively evaluate the health conditions of their babies and themselves. Thanks to such modified study design and comprehensive analytical methods, pivotal symptoms of PPD and the key influential factors were identified to provide evidence for improving the clinical practices for mothers and babies.

Admittedly, there are some limitations on this study. The survey respondents mainly consisted of urban-based women with vaginal deliveries, which meant our findings cannot be generalized to women with cesarean section or in rural areas. In addition, the maternal and infant outcomes were based on maternal self-report without a more objective evaluation, such as a clinical diagnostic interview. Finally, our PPD assessment was conducted within 6 weeks postpartum. It should be noted that the onset of some symptoms may occur beyond this period (Silva-Fernandez et al., 2023), and a later assessment might have yielded different results. These issues should be addressed in future studies, which could include randomized controlled trials to examine causal effects and longitudinal designs to track PPD symptoms over a longer postpartum period.

## 5 Conclusion

This study identified the central symptoms and important perinatal and postpartum factors that may trigger or exacerbate PPD. The high centrality of *Anxiety/Worry*, *Sad mood* and *Overwhelmed* in the depressive symptom network suggests that providing sufficient professional and family support to reduce those common early symptoms is critical for maintaining maternal mental wellbeing. The Bayesian network reflects the complexity of the interrelation between PPD and relevant factors, highlighting the importance of breastfeeding experience as well as maternal and infant health condition in the early postpartum period for PPD development. Our findings also revealed that hospital type served as a critical bridging node centrally embedded within the network. To effectively mitigate PPD risk and improve maternal mental health, policymakers must shift beyond facility-focused approaches by implementing evidence-based, humanized practices (e.g., nonpharmacy pain relief, prolonged SSC, tailored breastfeeding support) across all facilities, while simultaneously addressing upstream structural determinants that funnel vulnerability into specific care pathways. In conclusion, the network approach used in this study provides a more comprehensive insights into the important nodes and mechanisms underlying maternal mental health, which are of great value for health professionals to develop targeted interventions to prevent the development of PPD.

## Data Availability

The raw data supporting the conclusions of this article will be made available by the authors without undue reservation.
