# Supplementary material for: Network analysis of postpartum depression symptoms and perinatal risk factors in Chinese women: a prospective observational study
Source: Front Psychol. 2025 Nov 3;16:1604013. doi: 10.3389/fpsyg.2025.1604013 (PMC12620362; doi:10.3389/fpsyg.2025.1604013)
Supplement: Supplementary file 1 [file Supplementary_file_1.docx]

**Supplementary Table 1. The number of participants in each hospital**

| Hospital | Valid response at T1  n (%) | Valid response at T2  n (%) |
| --- | --- | --- |
| GH1 | 49 (9.8) | 35 (9.3) |
| GH2 | 47 (9.4) | 36 (9.5) |
| GH3 | 48 (9.6) | 47 (12.5) |
| MCH1 | 51 (10.2) | 29 (7.7) |
| MCH2 | 50 (10.0) | 47 (12.5) |
| MCH3 | 55 (11.0) | 40 (10.6) |
| MCH4 | 41 (8.2) | 24 (6.4) |
| MCH5 | 54 (10.8) | 48 (12.7) |
| MCH6 | 52 (10.4) | 29 (7.7) |
| MCH7 | 51 (10.2) | 42 (11.1) |
| Total | 498 (100.0) | 377 (100.0) |

Note. GH refers to general hospitals, MCH refers to Maternal and Child Health hospitals.

T1: 24­­–48 hours after delivery, T2: 4 to 6 weeks postpartum

**Supplementary Table 2. Detailed participant characteristics (n=377)**

| Variable | n | % |
| --- | --- | --- |
| Hospital type |  |  |
| General hospitals | 118 | 31.3 |
| Maternal & Child Health hospitals | 259 | 68.7 |
| Age group |  |  |
| <35 | 311 | 82.5 |
| ≥35 | 66 | 17.5 |
| Educational level |  |  |
| Junior college and below | 136 | 36.1 |
| Bachelor or above | 241 | 63.9 |
| Household registration |  |  |
| Both urban household register | 188 | 49.9 |
| Both rural household register | 98 | 26.0 |
| One urban household register | 91 | 24.1 |
| Annual household income  <120,000 yuan  120,000-300,000 yuan  >300,000 yuan | 128  155  94 | 34.0  41.1  24.9 |
| Living place at early postpartum |  |  |
| With husband | 87 | 23.1 |
| Husband and parents | 209 | 55.4 |
| Maternity care centers | 73 | 19.4 |
| Other | 8 | 2.1 |
| Primary postpartum caregiver |  |  |
| Husband | 214 | 56.8 |
| Mother/mother-in-law | 224 | 59.4 |
| Maternity matron | 176 | 46.7 |
| Parity |  |  |
| Primipara | 239 | 63.4 |
| Multipara | 138 | 36.6 |
| Pregnancy complication |  |  |
| Yes | 195 | 51.7 |
| No | 182 | 48.3 |
| Prenatal checkups |  |  |
| Regular | 342 | 90.7 |
| Irregular | 35 | 9.3 |
| Prenatal education |  |  |
| Yes | 134 | 35.5 |
| No | 243 | 64.5 |
| Mode of delivery |  |  |
| Spontaneous vaginal | 372 | 98.7 |
| Instrumental | 5 | 1.3 |
| Baby gender |  |  |
| Male | 206 | 54.6 |
| Female | 171 | 45.4 |
| Pain management |  |  |
| Pharmacy pain relief | 257 | 68.2 |
| Nonpharmacy pain relief | 245 | 65.0 |
| No pain relief method | 53 | 14.1 |
| Companionship |  |  |
| Prenatal companionship | 319 | 84.6 |
| Companionship during labour | 201 | 53.3 |
| Induced labour |  |  |
| Yes | 92 | 24.4 |
| No | 285 | 75.6 |
| Labour augmentation |  |  |
| Yes | 131 | 34.7 |
| No | 246 | 65.3 |
| Episiotomy |  |  |
| Yes | 88 | 23.3 |
| No | 289 | 76.7 |
| Duration of SSC (min) |  |  |
| No SSC | 63 | 16.7 |
| <30min | 154 | 40.9 |
| 30 – 59 min | 65 | 17.2 |
| 60 – 89 min | 21 | 5.6 |
| ≥90 min | 74 | 19.6 |
| Initiation of the first breastfeeding |  |  |
| Within 1 hour after birth | 298 | 79.0 |
| After 1 hour | 79 | 21.0 |
| Rooming in |  |  |
| Yes | 364 | 96.6 |
| No | 13 | 3.4 |

Note. SSC refers to Skin-to-Skin Contact

**Supplementary Table 3. Importance ranking of postpartum depressive symptoms in the network**

| Ranking | EPDS items | Degree | Betweenness |
| --- | --- | --- | --- |
| 1 | EPDS 4: Anxiety/worry | 0.893 | 0.888 |
| 2 | EPDS 8: Sad mood | 0.813 | 0.714 |
| 3 | EPDS 6: Overwhelmed | 0.673 | 0.714 |
| 4 | EPDS 1: Pleasure loss | 0.673 | 0.609 |
| 5 | EPDS 3: Self-blame | 0.428 | 0.000 |
| 6 | EPDS 5: Panic | 0.428 | 0.000 |
| 7 | EPDS 7: Insomnia | 0.428 | 0.000 |
| 8 | EPDS 9: Crying | 0.428 | 0.000 |
| 9 | EPDS 2: Pessimism | 0.428 | 0.000 |
| 10 | EPDS 10: Self-harm ideation | 0.000 | 0.000 |

Note. EPDS refers to the Edinburgh Postnatal Depression Scale

**Supplementary Table 4. The differences in perinatal care practices, CEQ scores, adverse health conditions and EPDS scores between general and MCH hospitals**

|  | General hospitals  n=118 (31.3%) | MCH hospitals  n=259 (68.7%) | χ^2^ | *P* |
| --- | --- | --- | --- | --- |
| Women with pregnancy complications |  |  | 16.067 | <0.001 |
| Yes | 43 (36.4) | 152 (58.7) |  |  |
| No | 75 (63.6) | 107 (41.3) |  |  |
| Blood loss ≥400ml within 2h after birth |  |  | 9.985 | 0.002 |
| Yes | 6 (5.1) | 44 (17.0) |  |  |
| No | 112 (94.9) | 215 (83.0) |  |  |
| Common infant health problems |  |  | 6.353 | 0.012 |
| Yes | 21 (17.8) | 78 (30.1) |  |  |
| No | 97 (82.2) | 181 (69.9) |  |  |
| Maternal health seeking behaviors |  |  | 1.694 | 0.193 |
| Yes | 22 (18.6) | 64 (24.7) |  |  |
| No | 96 (81.4) | 195 (75.3) |  |  |
| Breastfeeding challenges |  |  | 6.102 | 0.014 |
| Yes | 40 (33.9) | 123 (47.5) |  |  |
| No | 78 (66.1) | 136 (52.5) |  |  |
| Nonpharmacy pain relief |  |  | 16.954 | <0.001 |
| Yes | 59 (50.0) | 186 (71.8) |  |  |
| No | 59 (50.0) | 73 (28.2) |  |  |
| Pharmacy pain relief |  |  | 49.273 | <0.001 |
| Yes | 52 (43.2) | 206 (79.5) |  |  |
| No | 67 (56.8) | 53 (20.5) |  |  |
| Duration of SSC (min) |  |  | 32.819 | <0.001 |
| No SSC | 33 (28.0) | 30 (11.6) |  |  |
| <30min | 51 (43.2) | 103 (39.8) |  |  |
| 30 – 59 min | 23 (19.5) | 42 (16.2) |  |  |
| 60 – 89 min | 5 (4.2) | 16 (6.2) |  |  |
| ≥90 min | 6 (5.1) | 68 (26.3) |  |  |
| Childbirth experience (the mean CEQ-C score) | 2.96±0.44 | 3.37±0.44 | Z=-7.728 | <0.001 |
| Total EPDS score | 4.42±4.55 | 6.90±5.73 | Z=-4.023 | <0.001 |

Note. MCH refers to Maternal and Child Health, CEQ-C refers to the Chinese version of the Childbirth Experience Questionnaire, Z refers to Mann-Whitney U test
